# Computational simulation of cranial soft tissue expansion on the cranium during early postnatal growth in humans

**DOI:** 10.1111/joa.14211

**Published:** 2025-01-23

**Authors:** Amy Manson, Nathan Jeffery

**Affiliations:** ^1^ Department of Musculoskeletal & Ageing Science, Institute of Life Course & Medical Sciences (ILCaMS) and Human Anatomy Resource Centre (HARC), Education Directorate University of Liverpool Liverpool UK

**Keywords:** brain, cranium, geometric morphometrics, growth, integration, ontogeny, Procrustes

## Abstract

The importance of interactions between neighbouring rapidly growing tissues of the head during development is recognised, yet this competition for space remains incompletely understood. The developing structures likely interact through a variety of mechanisms, including directly genetically programmed growth, and are mediated via physiological signalling that can be triggered by structural interactions. In this study, we aimed to investigate a different but related potential mechanism, that of simple mechanical plastic deformation of neighbouring structures of the head in response to soft tissue expansion during human postnatal ontogeny. We use computational modelling and normative real‐world data to evaluate the potential for mechanical deformation to predict early postnatal cranial shape changes in humans. We test some aspects of the spatial packing hypothesis applied to the growing brain and masticatory muscles, and their effects on the cranium, with a particular focus on the basicranium and face. A simple finite element model of an early postnatal human cranium, brain and masticatory muscles was created from CT and MRI. Growth of the brain and muscles was simulated using a tissue expansion material. The effect of the expanding soft tissues on the cranium was assessed using geometric morphometrics, comparing the baseline model to simulation results, and also to normative cranial shape data collected from neonatal MRI (0–4 months of age). Findings revealed that cranial shape changes present in the normative sample were consistent with cranial base flexion and were largely allometric (size‐linked). Simulation of brain expansion produced broadly similar shape changes of the basicranium with most growth occurring in the cranial vault, while masticatory muscle expansion produced smaller and more widespread changes throughout the cranium. When simulated together, expansion of the masticatory muscles exerted a constraining effect on the results of brain expansion. Our findings that the simple growth simulations were able to mimic biological growth suggest that the relationship between regions of the developing head may be partly structural within the first few months of postnatal ontogeny in humans.

## Graphical abstract

We use computer simulations of soft tissue growth to investigate the role of simple physical deformation in the development of the postnatal human cranium. We found that simulated expansion of the model endocranial volume resulted in cranial shape changes consistent with those seen in a normative early postnatal sample, using Procrustes analyses. This suggests a largely structurally integrated relationship between regions of the developing head within the first few months of postnatal life.

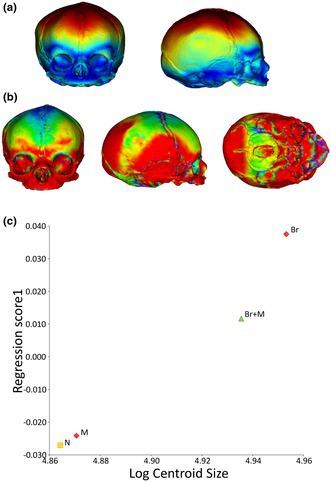

## 1 INTRODUCTION

The developmental period involves many complex interactions between often rapidly growing tissues. The differing spatial needs of growing organs and structures during development has been acknowledged at least as early as 1895, where Roux referred to ‘the struggle of the organs for space’ (Schumacher, 1997), subsequently referred to as ‘mechanically forced correlation’ by Gould and Lewontin (1979), which helped found theories of developmental integration and modularity (e.g. Cheverud, 1996; Jung et al., 2021; Ross & Ravosa, 1993; Zollikofer et al., 2017). However, while the interactions between neighbouring tissues are generally acknowledged as important, resulting in a phenotype reflective of collaboration between developmental regions, we lack sufficient empirical evidence on the hierarchical patterning of this competition for space.

Cranial postnatal development in modern humans is widely considered to be driven by our enlarged brains (Barbeito‐Andrés et al., 2015; Liang et al., 2023; Zollikofer et al., 2017), while our recent paper (Jeffery & Manson, 2023) suggests that postnatal cranial ontogeny in other primates, such as macaques, may be more closely aligned with masticatory muscle and facial growth. The masticatory apparatus of humans has diminished over evolutionary time, likely relating to behavioural adaptions concerning diet (Zollikofer et al., 2022), alongside the downregulation of myosin heavy chain in the *Homo* genus leading to smaller, weaker, more gracile masticatory musculature compared to that of other extant primates and fossil hominids (Stedman et al., 2004). Variations within and co‐variations between these key elements of the head region, namely the brain, the masticatory muscles and the intervening skull, can shed light on the competition for space between tissues during growth and development. In the current paper, we use the term ‘ontogenetic variation’ to mean growth or change along a trajectory, and ‘intraspecific variation’ to describe differences between individuals at the same age or same morphogenetic stage.

### 1.1 Ontogenetic variation

Brain growth during early postnatal ontogeny in modern humans is remarkable. Overall cerebral and cerebellar volumes double within the first year, reflecting, for example, the rapid development of motor coordination and balance in infants (Knickmeyer et al., 2008). Brain size continues to increase, albeit at a slower rate, for the next 2 years, and the rate of brain growth slows again after around 3 years of age (Barbeito‐Andrés et al., 2020), approaching adult brain volume (~90%) by the age of 5–6 years postnatally (Giedd, 2004; Reiss et al., 1996), while growth of the face and cranial base continues at a more gradual pace (Zollikofer et al., 2017, 2022).

During early postnatal life, the bones of the cranial vault are unfused resulting in the morphology of the calvarium largely reflecting that of the rapidly growing brain (Zollikofer et al., 2017). The newborn cranium is approximately 25% adult size, doubling in the first 6 months (Libby et al., 2017; Mercan et al., 2020). The cranial vault approaches adult size (~90%) at around the same age as the brain, roughly 5 to 6 years postnatally (Opperman et al., 2005). Brain growth is considered to be an important driver of cranial growth and development during this period (Weickenmeier et al., 2017). The majority of postnatal development of the cranium is allometric, with different structures presenting with different growth patterns, and little evidence of non‐allometric shape changes (Sardi et al., 2007). The cranial vault and basicranium, both located in close physical proximity to the brain, covary strongly during early postnatal ontogeny, largely driven by the rapid neural expansion (Barbeito‐Andrés et al., 2015). The face initially grows at a slower rate than the basicranium, with both regions continuing to increase in size steadily beyond the cessation of brain growth, varying more gradually and for longer during ontogeny, until around 13 years and 15–16 years of age for the basicranium and face, respectively (Bastir et al., 2006; Lieberman et al., 2000; Opperman et al., 2005; Wellens et al., 2013).

In most mammals, masticatory musculature undergoes a phasic change during ontogeny similar to that of the brain and skull. Muscles of mastication mature later than other skeletal muscles, such as those of the tongue, and are not fully developed at birth (Yamane, 2005). In prenatal life, masticatory muscles generally produce some movement but are not yet functional in the sense of feeding. Later during the neonatal and infant stages, milk is ingested by suckling requiring specific jaw and mouth movements, followed by a gradual transition to mature masticatory movements associated with weaning. This is followed by growth and cellular differentiation of the masticatory muscles during the juvenile period (Herring, 1985). Development of human masticatory muscles has been found to follow a largely similar growth trajectory among the muscles (masseter, temporalis, medial and lateral pterygoid) (Sato et al., 1994) with some slight differences reported between temporalis and masseter (Dickinson et al., 2018). Measurements of human masticatory muscles during prenatal and postnatal ontogeny are scarce, and to the best of the authors' knowledge, no studies have been carried out on masticatory muscles in modern human neonates. However, in other primates such as macaques, masticatory muscle growth follows a linear trajectory through infancy and the juvenile phase (Jeffery & Manson, 2023).

### 1.2 Ontogenetic co‐variation

These trends of ontogenetic variation do not occur in isolation, particularly in complex and tightly packed regions such as the head. As structures develop, they may influence or be influenced by other nearby developing structures. This ontogenetic co‐variation may take the form of genetically determined growth; biomechanical interactions involving flexibility and compliance of the cranial cavity and bones; physiological interactions such as local paracrine cell signalling, potentially mediated by the intervening meningeal layers; and structural interactions where growth of the brain and other soft tissues directly influence cranial form (Lesciotto & Richtsmeier, 2019).

Evolutionarily, an increase in encephalisation incurs significant metabolic cost on the organism, which may result in a trade‐off giving rise to decreased skeletal muscle mass and a smaller gastrointestinal tract in modern humans compared with other primates as a means of maintaining reasonable metabolic requirements and energy expenditure (Isler & Van Schaik, 2014; Muchlinski et al., 2018; Navarrete et al., 2011). This metabolic trade‐off between brain and body mass appears to be mirrored in modern human ontogeny with an inverse relationship between peak brain growth and body growth rates (Kubera et al., 2013; Kuzawa et al., 2014). According to Biegert's spatial packing hypothesis (1963), an evolutionary increase in encephalisation results in increased basicranial flexion as a means of accommodating the expanding brain. This may have implications for ontogenetic development and brain growth.

Postnatal growth of the cranial vault occurs by a combination of expansion of calvarial bones via sutural growth and modelling, and by displacement of these bones by the growing brain (Galea et al., 2021; Jin et al., 2016), while growth of the cranial base is by bone modelling, and expansion at synchondroses. During early postnatal life, the bones of the cranial vault are unfused resulting in the morphology of the calvarium largely reflecting the morphology of the rapidly growing brain (Zollikofer et al., 2017). Brain expansion may, through mechanical pressures exerted on the cranial bones and membranes, trigger osteoblasts to proliferate, differentiate and produce extracellular bone matrix, thereby mediating growth and expansion of cranial bones, although the precise mechanism is not well understood (Barbeito‐Andrés et al., 2020; Henderson et al., 2004; Weickenmeier et al., 2017). The compressive and tensile stresses on the bone exerted by the adjacent expanding brain, which may be transmitted via the meninges through mechanical loading, may induce ossification centres during early development (Galea et al., 2021) and may subsequently reshape the cranial bones by localising osteoblasts and osteoclasts in areas of higher and lower tensile stress, respectively (Edamoto et al., 2019; Weickenmeier et al., 2017).

Growth of the masticatory muscles has been shown to have an effect on face shape and size, and also to influence parts of the cranial base, and the mandible (Kiliaridis, 1995; Scott, 1954; Sella‐Tunis et al., 2018). The action of masticatory muscles may shape the cranium by influencing cranial sutural growth (Behrents et al., 1978), by thickening and reshaping cortical bone through generation of tensile stresses (Scott, 1954), by increasing vascularity to the bone near muscle attachment sites (Kiliaridis, 1995) or through constraining effects relating to structure and function. For example, temporalis muscle shapes the cranial vault in some primates through the strength and scaling relationships of the muscle attachments, which create sagittal crests as a means of increasing the attachment area available for this muscle. The vertical repositioning of the vault walls in the modern human cranium gives a better alignment for the temporalis muscle and tendon, leading to increased mechanical efficiency which allows for a relative decrease in muscle size and strength, and reduces the need for the additional muscle attachment sites seen in other primates such as gorillas (Oyen, 1997). Cranial base flexion decreases with increasing size of the masticatory apparatus, according to Biegert's (1963) spatial packing hypothesis, due to the increasing spatial constraint of larger masticatory muscles anterior to the cranial base. Masticatory apparatus growth has been suggested to constrain brain growth, and has additionally been proposed to constrain the effects of brain growth on the cranium (Jeffery et al., 2021).

Integration and interactions between developing structures of the postnatal human head may take the form of, for example, functional integration which allows development and growth of structures without compromising their functionality; or developmental integration where elements interact during their formation or are directed by a common systemic source such as a circulating hormone. In addition to these mechanisms for patterns of covariation, simple mechanical plastic deformation may also be important, whereby adjacent neighbouring structures directly physically influence each other through their physical proximity and growth. Simple plastic deformation has been shown to predict cranial shape changes in response to increasing demands of soft tissue expansion through computer simulation in mice (Jeffery et al., 2021). This raises the question—can simple plastic deformation also predict a substantive part of the cranial shape variations seen in modern humans during postnatal life, or are these changes more closely related to non‐structural interactions? Here, we evaluate the potential of simple mechanical deformation to predict changes of cranial shape during early postnatal ontogeny in humans. We test aspects of the spatial packing hypothesis applied to the growing brain and masticatory muscles, and their varying effects on the cranium, with a particular focus on the basicranium and face.

- Objective 1—Map normative shape changes across a population of neonates ranging from 0 to 4 months of age. This will then form the framework against which to compare the predictive power of the computational simulations. These data represent a myriad of mechanisms of integration as well as many other sources of variation and variability, not just plastic deformation.
- Objective 2—Measure normative volumetric changes of the brain and the masseter (representing the masticatory muscles) within a subset of the population of neonates, comparing volumes for individuals of around 14 days and roughly 3 months of age. This will inform the scale of volumetric changes that will be computationally simulated.
- Objective 3—Run 3D simulations based solely on the principle of tissue deformation driven by volumetric soft tissue expansion defined within Objective 2 and compare simulated shape changes with real‐world changes defined within Objective 1.

## 2 METHODS

### 2.1 Sample data

**FIGURE 1:**
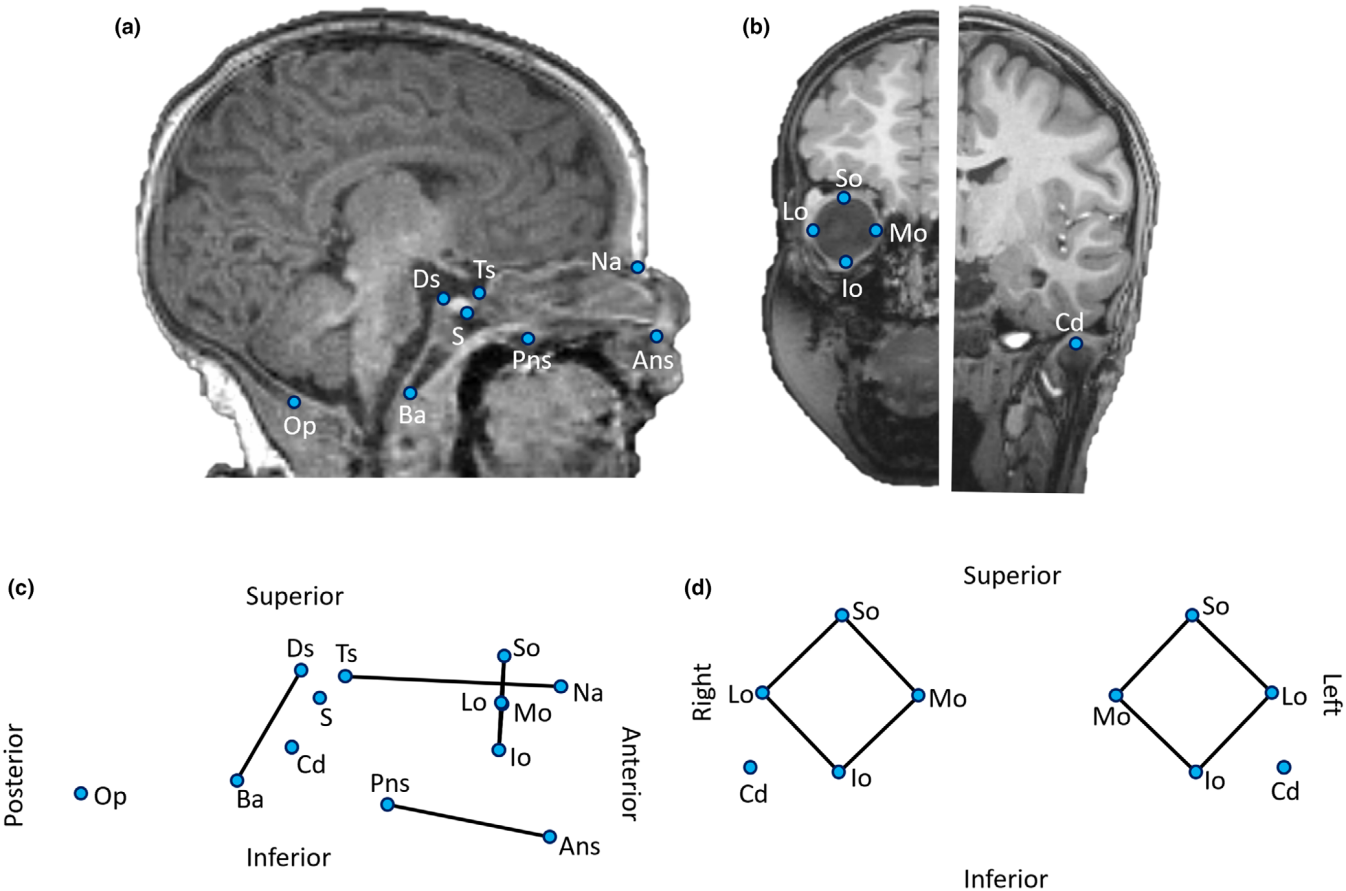
Example T1‐weighted MRI showing landmark placement. (a) Midsagittal image; (b) offset half coronal images. Landmark configuration and wireframe from (c) lateral view; (d) frontal view. Ans, anterior nasal spine; Ba, basion; Cd, condylion; Ds, dorsum sellae; Io, inferior orbital margin; Lo, lateral orbital margin; Mo, medial orbital margin; Na, nasion; Op, opisthion; Pns, posterior nasal spine; S, sella turcica; So, superior orbital margin; Ts, tuberculum sellae.

Sample data were used to establish baseline volumetric expansions in order to inform the model simulations, and also to act as a framework against which the simulation results could be compared. To provide a real‐world framework for Objective 1, 3D coordinates for a configuration of 18 readily identifiable bony landmarks of the basicranium and face (see Figure 1) were captured using the markups tool in 3D Slicer v4.11.2 (Fedorov et al., 2012) directly from T1‐weighted MRI scans for individuals between 0 and 4 months of age (*n* = 62). These were obtained from the NIMH Data Archive (https://nda.nih.gov/) *n* = 42; and from the ALBERT dataset (Gousias et al., 2012, 2013), *n* = 20. Landmarks were selected to provide reasonable morphological representation while minimising the landmarks‐to‐individuals (*p*/*n*) ratio (see Bookstein, 2017, 2019; Cardini, 2018; Cardini et al., 2019). Landmarks on the calvarium were difficult to accurately and repeatably place because of the similar MR signal yields between the thin and partially unossified calvarial bones and the surrounding soft tissues, and were therefore not included in the collection of normative data. The same observer (AM) carried out all landmark placements. Intraobserver landmark repeatability was tested by placement of the anatomical landmark set three times each for five representative individuals over the course of several days. At the time of submission, the secondary analyses of fully anonymised human imaging data do not require University of Liverpool ethics approval.

Basicranial‐facial centroid size was calculated as the square root of the sum of squared distances between the landmarks shown in Figure 1. Geometric morphometric allometric (size‐related) variations of the configuration of landmarks were investigated in MorphoJ (v1.07a) using multivariate regressions of symmetric Procrustes coordinates against log‐transformed centroid size. Residuals from this regression were used to explore non‐allometric shape variation. Warped surfaces were created to illustrate basicranial‐facial form variation within the normative sample population, with reference to the co‐ordinates generated by MorphoJ, using Landmark v3.0 (Institute for Data Analysis and Visualization (IDAV) and the University of California, Davis).

**FIGURE 2:**
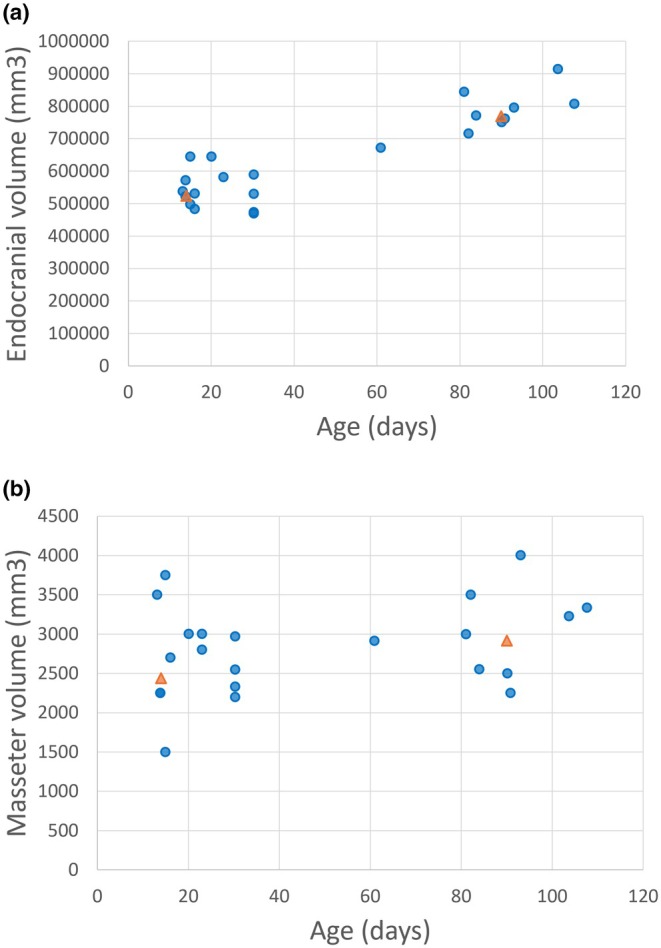
Plots showing (a) measured endocranial volume (EV, mm^3^) and (b) measured masseter volume (mm^3^), blue dots; alongside calculated average volumes at 14 days and 90 days for each (orange triangles).

**FIGURE 3:**
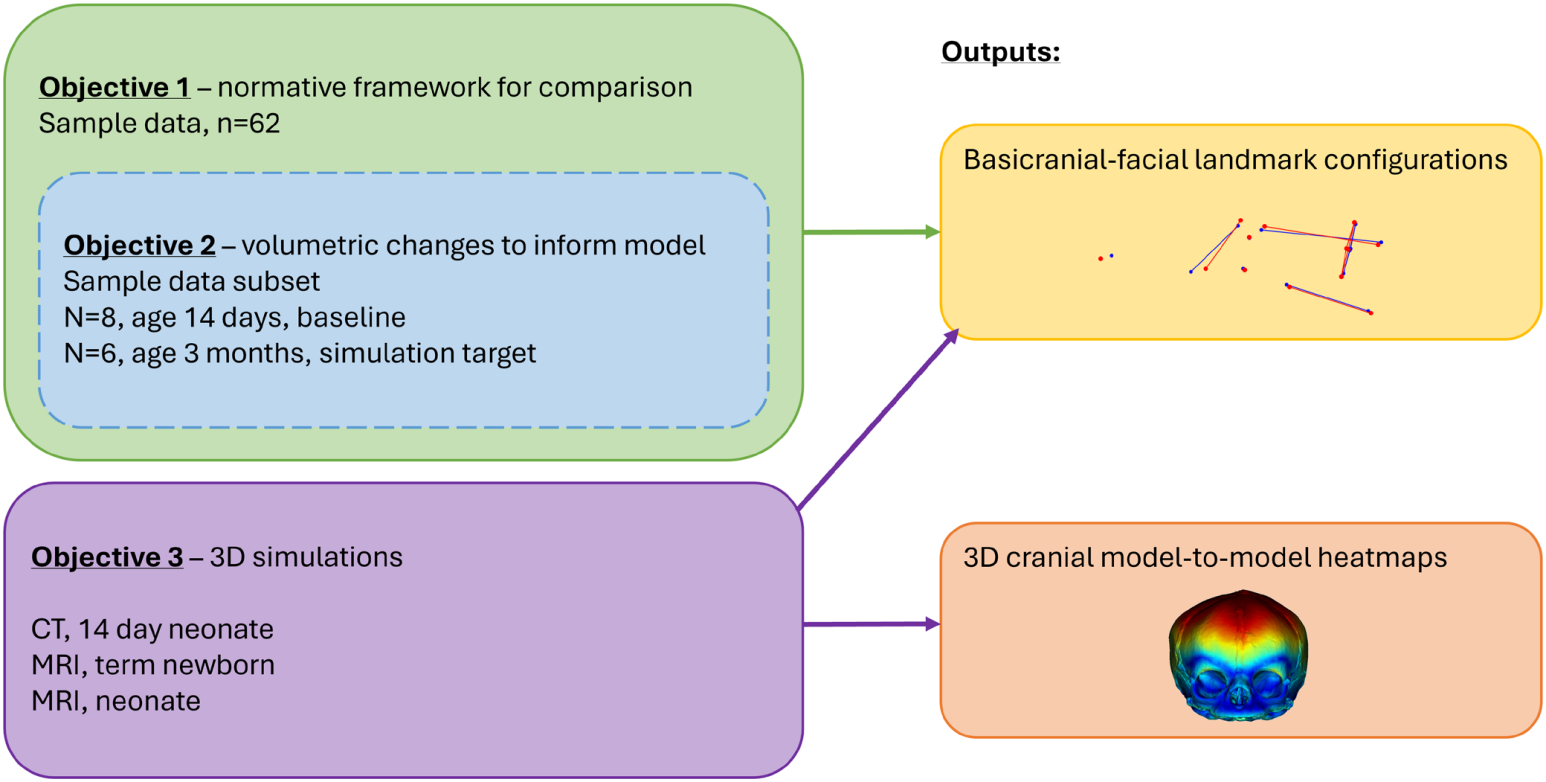
Graphic illustrating the data used within each objective of the study, and outputs from each objective. Landmark configurations were obtained using MorphoJ, while 3D model‐to‐model heatmaps were produced from the baseline model and simulation results in 3D Slicer.

For Objective 2, volumetric data for masseter muscle and the endocranium for a subset of the sample data were collected and subsequently used to inform the computational model muscle and brain expansions (see Figure 2). These were calculated using the stereological method implemented via Volumest plugin (Merzin, 2008) for ImageJ v1.53k (Schneider et al., 2012). Endocranial volume is reliable as a proxy for brain size in many species, including mammals (Dumoncel et al., 2021; Iwaniuk & Nelson, 2002; Pengas et al., 2009). Averages for endocranial volume and masseter muscle volume were calculated for all scans between the ages of 12 and 16 days (*n* = 8) to establish baseline volumes of similar age to the neonatal skull used as the baseline for the computational model. Averages were also calculated for all scans between the ages of 88 and 92 days (*n* = 6), to act as a target volume for deformation of the model. See Figure 3 for a schematic summary of the data used within each objective of the study. These figures were subsequently used as target volume changes in the computational simulation of early postnatal cranial soft tissue growth in humans, described below. The aim of the simulation was to investigate direct plastic deformation resulting from the expansion of these soft tissues.

**FIGURE 4:**
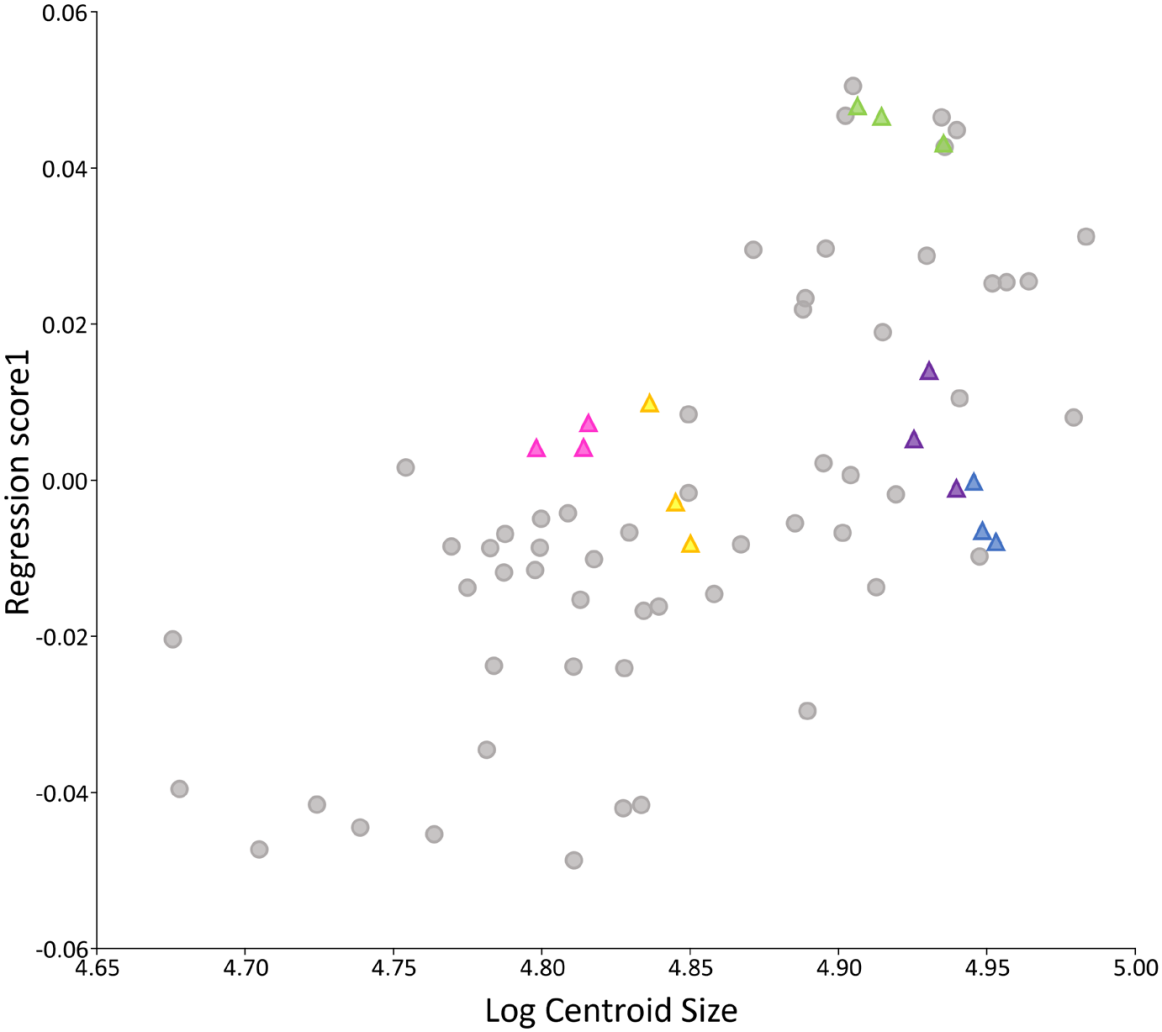
Regression plot of basicranial‐facial shape against log centroid size. Data points of repeated landmark configurations represented by coloured triangles. Other landmark configurations within the dataset are grey dots.

Landmarking repeatability was assessed visually by analysing the repeat landmark configurations together with the sample data in several key graphs to indicate the significance of shape variation from landmarking imprecision compared to biologically meaningful shape variation across the study cohort (Jeffery & Spoor, 2004). Repeated landmarking configurations of the same individuals were located relatively close to one another on plots, confirming that intraobserver error or variation in landmark placement was less than interindividual variation in the study cohort. See Figure 4.

### 2.2 Computational simulation

**FIGURE 5:**
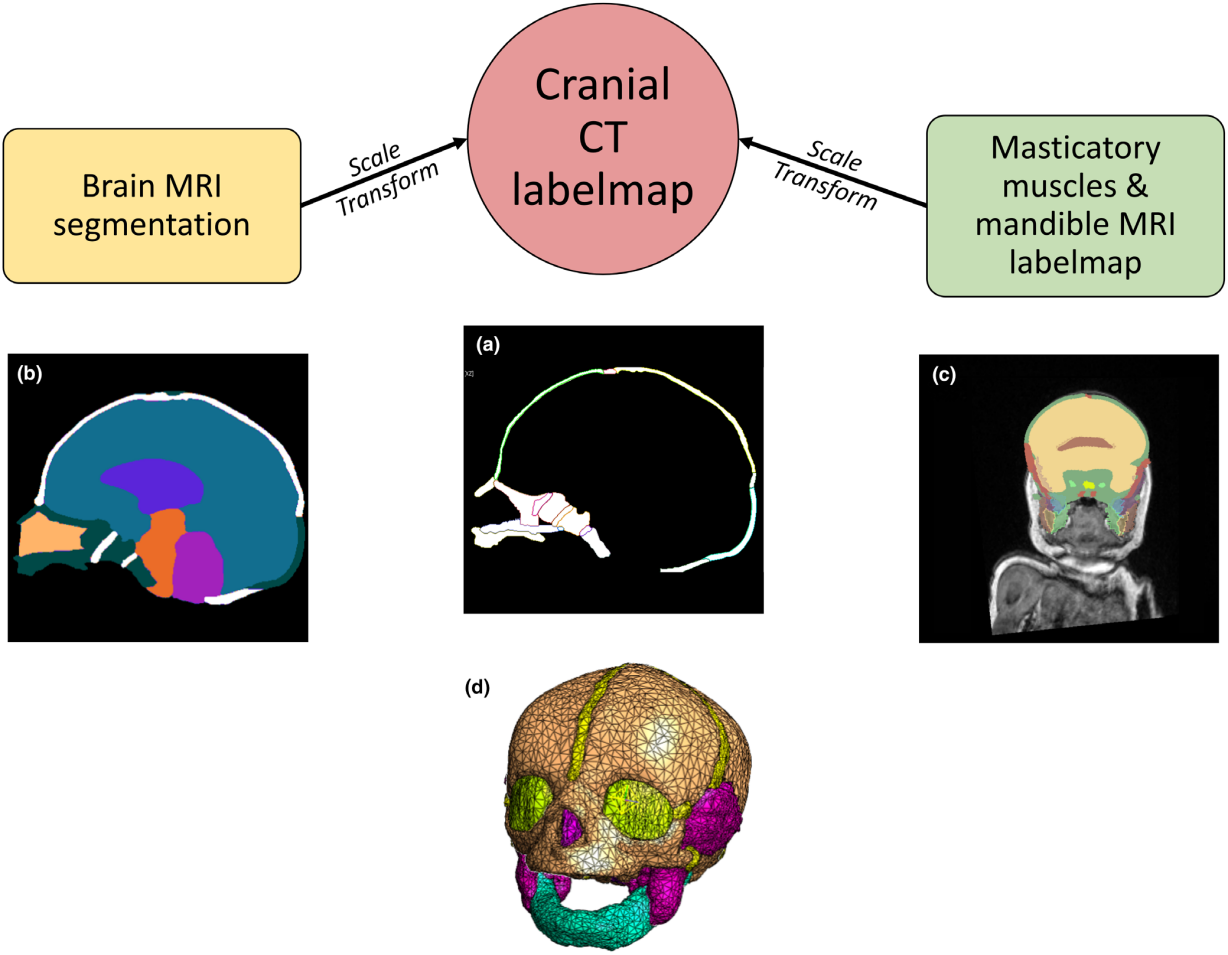
Both MRI‐derived datasets were scaled and transformed to fit the CT data. (a) A model of a neonatal cranium was created in Amira v5.4.0 (Stalling et al., 2005) from a CT scan of a 19‐day‐old dry museum specimen (nr10) from a 19th century collection (University of Vienna). Bones of the cranium, cranial sutures and synchondroses, and nasal cartilage were segmented in Amira using threshold segmentation. (b) A brain model was created using a brain segmentation labelmap provided with the ALBERT dataset (ALBERT1) (Gousias et al., 2012) representing a neonate at term, then scaled and transformed to fit the CT data in Amira. (c) The created cranium‐brain labelmap was then merged with a T1‐weighted MRI of a neonate from the NIMH data repository (NDARAE381RLK) in 3D Slicer v4.11.2 (Fedorov et al., 2012), again transforming and scaling the MRI to fit the cranial CT spatial coordinates system. Masticatory muscles (masseter, temporalis, lateral and medial pterygoid) were manually segmented in 3D Slicer using the MRI as reference. The mandible was also manually segmented to provide a natural attachment point for the muscles of mastication. (d) The final volumetric tetrahedral mesh was generated in Amira.

For Objective 3, plastic deformation of the cranium due to endocranial and masticatory muscle enlargement was simulated in‐silico using a finite element approach in FEBio Studio v1.6.0 (Maas et al., 2012), utilising cell growth material which is based on mass exchange gradients (see Ateshian et al., 2009, 2012). A composite model representing skull, brain and masticatory muscles was created by merging three datasets: CT of a neonatal cranium sdry museum specimen nr10, University of Vienna; (Neubauer et al., 2009) to represent the bony elements; an existing brain segmentation labelmap of a neonate (ALBERT1, Gousias et al., 2012); and T1‐weighted MRI of a neonate showing masticatory muscles and mandible (NDARAE381RLK, Fedorov et al., 2012). Both the brain segmentation and MRI were scaled and merged with the CT dataset, which had the highest voxel resolution. Refer to Figure 5 for a visual representation of the process.

The merged datasets were used to create a model of the skull, brain and masticatory muscles in Amira v5.4.0 (Stalling et al., 2005). The labelmap was expanded and simplified to retain the essential geometries of the tissues of interest while minimising computational instabilities that can arise from small and irregular details such as cranial nerve foramina and turbinates. The labelmap was converted to a tetrahedral mesh in Amira. The final mesh consisted of 40,000 faces representing 212,000 tetrahedral elements and was imported into FEBio Studio for parameterising and solving.

For the purposes of this study, skull elasticity was set as invariant spatially as well as for the duration of the simulation. A Young's modulus of 300 MPa was used to achieve a stable deformation in response to soft tissue expansion. For simplicity, growth of soft tissues was assumed to be largely homogeneous in the present study. Other elements such as cranial meninges, cerebrospinal fluid, and cervical skeletal and muscular elements were omitted to focus on the key deformation hypothesis. The occipital condyles were each used as rigid fixed constraints, with a third acting as a spatial anchor within the diencephalon. Mass exchange gradients of the cell growth material were adjusted to achieve approximately the target volumetric changes of +60% and +35% for brain and masticatory muscles respectively. The mathematics of this material are described in Ateshian et al. (2009, 2012). In this simulation, intracellular solid volume (*phir*) of the expanding soft tissue varied from 0.3 to 1.35, and from 0.3 to 0.9 for the brain and muscle growth simulations respectively. The number of moles of membrane‐impermeant solute within the cell (*cr*) varied from 210 to 945, and from 210 to 630 for the brain and muscle growth simulations respectively. Extracellular osmolarity (*ce*) was set at 300 for all simulations and remained unchanged through simulation time. Due to the complexity and non‐linearity of the model, it was not possible to arrive at a specific target value, so ultimately the model was expanded by +69% and +32% for brain and masticatory muscles respectively. See Table 1 for the actual percentage increases achieved in the model for each growth simulation.

**TABLE 1:** Computationally driven changes of volume based on neonatal baseline (N) mesh.

Description	Simulation ID	Δ endocranial volume %	Δ muscle volume %
Neonatal baseline model	N	0	0
Brain growth	Br	+69	0
Muscle growth	M	0	+32
Brain + muscle growth	Br+M	+69	+32

In order to simulate growth of the brain (Br), the baseline model (N) was elastically deformed to accommodate a computationally driven 69% increase of endocranial volume. This was repeated for growth of masticatory muscles (M) which were expanded by 32%. The remaining simulation (Br+M) was used to investigate the relative influences of the expanding brain and muscles on the baseline model (see Table 1). The simulations were solved using a nonlinear method, then the results were landmarked and incorporated into the Procrustes shape analyses of the normative dataset. Whole mesh deformations were also visualised in FEBio, with 3D heatmap visualisations of the model baseline and results created in 3D Slicer using the model‐to‐model distance function. The heatmap visualisations allowed assessment of shape changes occurring across the whole cranium between the baseline model and simulation result. These whole model comparisons should not be confused with the landmark suites used to explore differences of the basicranium and face shape between models and the normative real‐world data (see above).

## 3 RESULTS

### 3.1 Sample data

**FIGURE 6:**
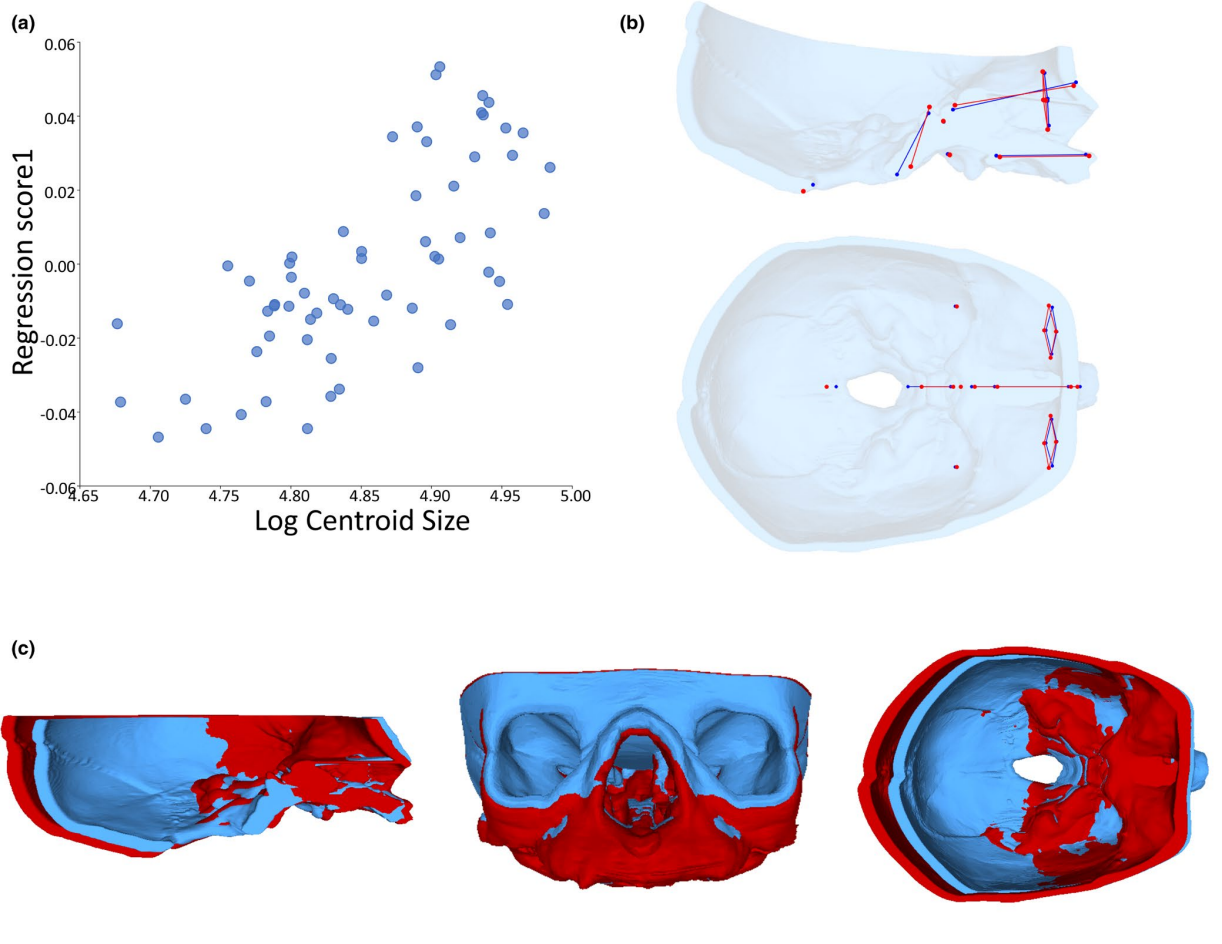
Allometric (size related) shape changes of basicranial‐facial shape: (a) plot of regression scores from the Procrustes form space against log centroid size; (b) wireframes (lateral and superior views) representing the allometric trend from smallest individual (blue) to largest individual (red). Scale ±0.15. (c) 3D surface warps of the landmarking results (blue = smaller, red = larger), midsagittal, anterior and superior views (cranial vault removed). No calvarial landmarks were used due to difficulties with accurate and repeatable placement.

Regressions of the symmetric component of the Procrustes coordinates (*n* = 62) against size (log centroid size) of the basicranial–facial complex for the real‐world data revealed allometric shape changes within the first 4 months of postnatal life consistent with a relative lengthening of the posterior cranial fossa and increasing cranial base flexion as overall size increased, illustrated in Figure 6 Maas et al., 2012. Size explains approximately 5% of the total allometric shape variation in the sample, *p* = 0.0018, with age predicting a similar 6% of total allometric shape variation, *p* = 0.0001. Non‐allometric analysis using the residuals from the regression of shape against log centroid size revealed that the majority of the age‐related shape variation was aligned with size, with the regression of non‐allometric shape against age predicting only 0.8% of total non‐allometric shape variation. Using volumetrics, brain volume was estimated to increase by around 60% between 14 days and 3 months of age, while masseter muscle volume increased by approximately 35%. The changes of volume were used to inform the 3D tissue deformation simulations of Objective 3.

**FIGURE 7:**
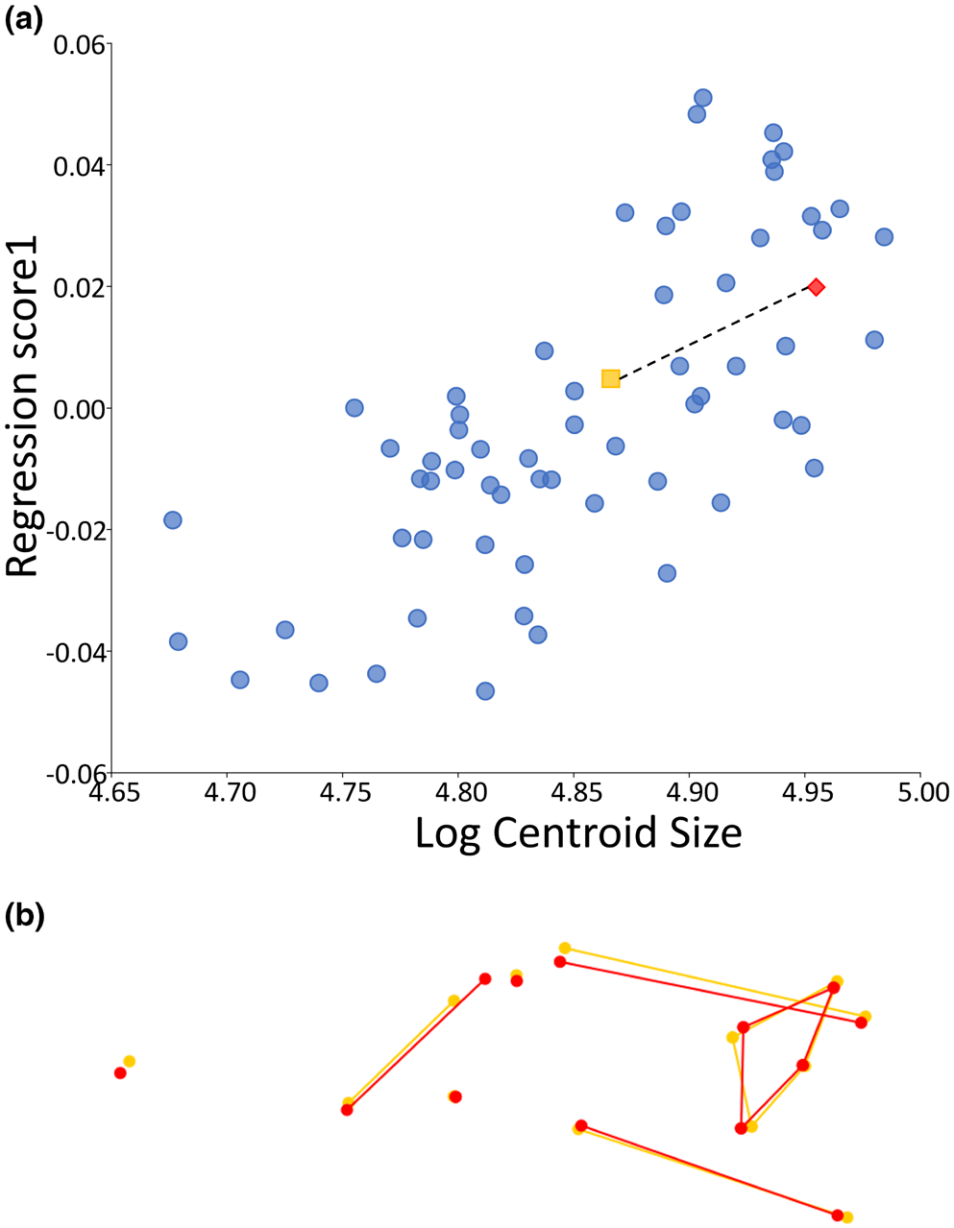
Allometric basicranial‐facial shape changes between baseline and brain growth simulation model (Br): (a) regression scores from the Procrustes form space against log centroid size including neonate baseline model (N, yellow square) and brain growth simulation (Br, red diamond) based on basicranial‐facial landmarks only. Real‐world data represented by blue dots. Dotted line illustrates change between baseline and growth models illustrated by (b) wireframes representing variation in basicranial‐facial landmark configuration between baseline (N, yellow) and brain growth simulation (Br, red). Scale ±0.5.

**FIGURE 8:**
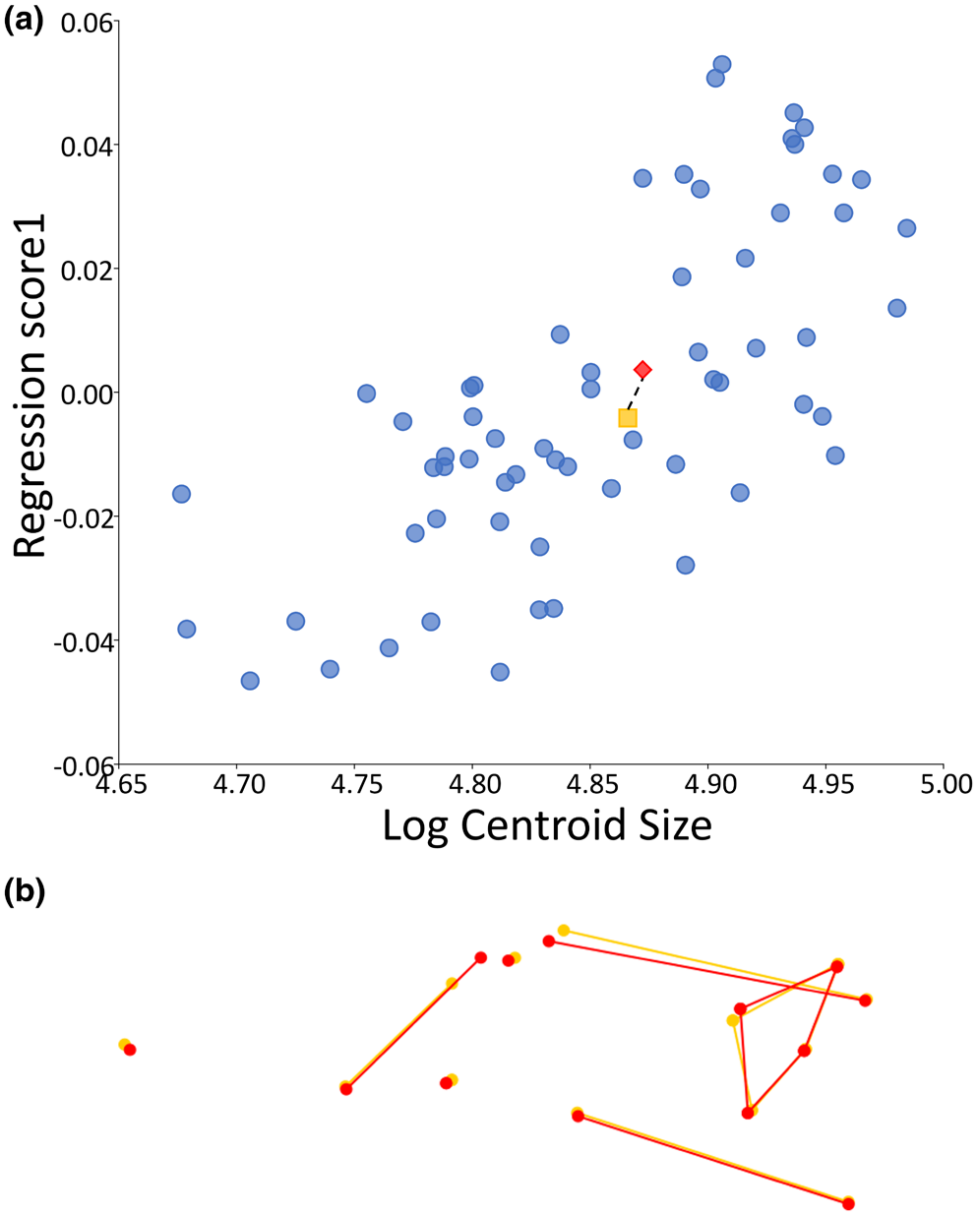
Allometric basicranial‐facial shape changes between baseline and masticatory muscle growth simulation model (M): (a) regression scores from the Procrustes form space against log centroid size including neonate baseline model (N, yellow square) and muscle growth simulation (M, red diamond) based on basicranial‐facial landmarks only. Real‐world data represented by blue dots. Dotted line illustrates change between baseline and growth models illustrated by (b) wireframes representing variation in basicranial‐facial landmark configuration between baseline (N, yellow) and muscle growth simulation (M, red). Scale ±0.5.

**FIGURE 9:**
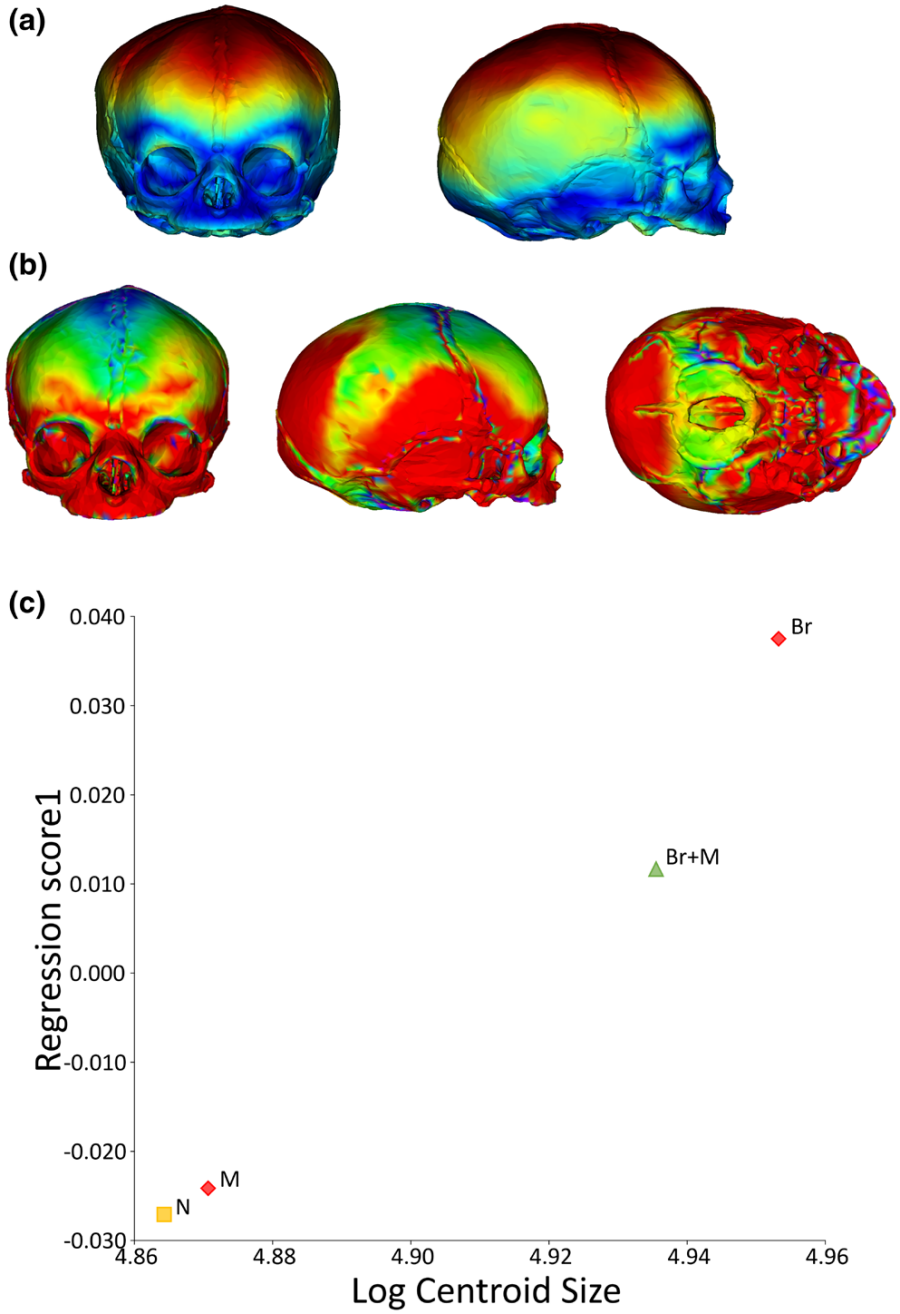
3D model‐to‐model heatmap visualisations generated from direct comparison between baseline neonate model (N) and simulation result for (a) brain growth simulation (Br), and (b) muscle growth simulation (M), representing comparison of shape across the whole cranium. Red = more growth, blue = less growth. (c) Allometric craniofacial shape changes between baseline and growth simulation models: regression scores from the Procrustes form space against log centroid size including neonate baseline model (N, yellow square), simulations of brain growth (Br), masticatory muscle growth (M), combined brain and muscle growth (Br+M), red diamonds.

### 3.2 Computational simulation

Computational simulations are summarised in Table 1. To evaluate our approach, the regression of basicranial‐facial shape against size described above (Figure 6) was repeated for each simulation. Each regression included the normative sample dataset alongside landmark configurations for the basicranial‐facial region of the baseline neonate model (N) and one of the simulation results (Br, M). Figures 7 and 8 show the regression plots for the symmetric shape component against size of the landmark configuration for the model baseline and result alongside the normative data framework, with accompanying wireframes illustrating shape change between the basicranial‐facial region of the baseline model (N) and growth simulation model (Br, M). The simulated 69% brain expansion (Br) resulted in a large change in size of the basicranial–facial complex but a relatively small change in shape of this region compared to the baseline neonate. The difference in regression score between baseline and brain expansion simulation was around 15% of the total variation in the sample (refer to Figure 7a). Analysis of the landmark configuration revealed that shape change seen between the model baseline and brain growth simulation result in the region of the face and cranial base included a relative lengthening of the posterior cranial base, a deepening and lengthening of the posterior cranial fossa and relative ventral shift of the anterior cranial base. Simulated enlargement of the masticatory muscles by 32% resulted in only a small change in both shape and size of the basicranial–facial complex in comparison to the baseline. The difference in regression score between baseline and masticatory muscle expansion simulation was around 8% of the total variation in the sample (refer to Figure 8a). Figure 9 shows the 3D cranial heatmap visualisations which illustrate the overall shape change from the baseline neonate model to each simulation result (Br, M). The heatmap visualisations allow comparison of deformation changes occurring in the simulations across the whole cranium. From analyses of the heatmap visualisations, the majority of form change associated with the brain growth simulation (Br) occurred in the region of the cranial vault, with relatively less change seen in the face and cranial base. Although deformations were smaller in the masticatory muscle growth simulation (M), they were widespread with small amounts of expansion occurring in the face, anterior cranial base and occipital regions.

The baseline neonate model (N), simulations (Br, M), and their combined iteration (Br+M) were then compared in a single shape space, omitting the normative sample dataset (Figure 9c). The landmark configuration for simulated masticatory muscle expansion (M) remained close to the baseline (N), while the simulation of brain expansion (Br) showed a large change in both shape and size of the landmark configuration. The combined iteration (Br+M) demonstrated an intermediate position between simulations of brain and muscle expansion, suggesting that expansion of the masticatory muscles may act as a constraint on the effects of brain expansion in this simulation.

These findings confirmed that simulations broadly mimic actual shape variations observed within this growth series and indicated that simulated endocranial growth had the greatest influence on both size and shape of the areas studied.

## 4 DISCUSSION

This study aimed to create a stable model of deformation of the human neonatal cranium that was driven towards target volumetric changes of cranial soft tissues. The resulting cranial shape changes were compared with a normative real‐world sample aged between 0 and 4 months of age using Procrustes analyses. Our main finding was that simulated expansion of the model endocranial volume resulted in cranial shape changes consistent with those seen in the normative sample. Here we discuss our main findings in the context of previous research.

As expected, most deformation seen in the brain growth simulation (Br) occurred in the area of the cranial vault, which correlates well with known early growth patterns of the neonatal cranium and brain where brain growth is one of the main drivers of form change of the calvarial bones during early postnatal ontogeny (Bruner, 2015; Jeffery et al., 2022; Weickenmeier et al., 2017). Shape change of the cranial base and face was less pronounced than that of the cranial vault in the brain growth simulation. Patterns of shape change in the basicranium and face largely corresponded with those observed in the real‐world normative data—the posterior cranial base lengthened relative to the rest of the landmark configuration, the posterior cranial fossa deepened and the anterior cranial base was shifted ventrally which may suggest basicranial flexion. The simulation of brain growth (Br) aligned well with the real‐world data in regression analyses, suggesting that growth of the cranial base and face may be driven by growth of the brain within the first few months of postnatal life. The masticatory muscle growth simulation (M) resulted in small amounts of deformation which were widespread across the cranium. The brain growth simulation (Br) resulted in the greatest change in size of the model basicranial–facial complex through simple plastic deformation of the modelled bones, while combined brain and muscle growth (Br+M) led to an intermediate change in size of this region of the cranium between brain and muscle growth simulations. This suggests a potential constraining role of the expanding muscles on the effects of brain growth in this simulation, similar to that previously noted in the mouse (Jeffery et al., 2021) and in the macaque (Jeffery & Manson, 2023).

In our growth simulations, the expanding brain had the greatest influence on both size and shape of the cranial base and face of this finite element model. The brain growth simulation (Br) fit well within the normative data, which indicates that within this period of ontogeny (roughly the first 3–4 months of postnatal life) form changes of the cranial base and face may be largely due to the structural relationship of regions of the head (brain and basicranial–facial complex) and that these structural interactions may be important for cranial shape changes.

The small amount of total shape variation of the basicranial–facial complex in the normative sample which was explained by size (5%) and age (6%) in this present study could indicate a degree of individual variation which may be due to underlying population genetics driving genetically determined growth during early development. The observed small per cent of explained shape variation in the context of growth may be as a result of a large number of growth processes occurring simultaneously during this period, and/or the relatively small scale of this analysis. The ontogenetic period studied here is dominated by cerebral expansion, but because the landmarks used in this study do not include the calvarium, the overall size effect observed is much smaller than overall cranial growth, with only a ~20% increase in centroid size from smallest to largest individuals in this basicranial‐facial region compared to the measured ~70% increase in brain volume within the same sample. A larger scale of size change may result in a stronger association between shape and size. During later postnatal development, increasing functional demands of structures of the head may become increasingly prominent. Craniofacial forms may diverge with time due to variations in timing of events such as fusion of cranial sutures and synchondroses; tooth eruption or loss; differences of diet due to weaning; and other factors. In the current study, similar shape changes were seen in the cranium with increasing size as with age, suggesting that shape changes of the cranium are tightly linked with growth of the head during early postnatal development. The remaining shape variation (~95%) which is not accounted for by size and age shows the diversity of growth and development driven by a range of factors, including biological sex, ethnicity, socioeconomic background, maternal health, nutrition, genetics, feeding regime, exposure to chemicals and infections and others. Additionally, some of this remaining variation may be reflective of some degree of noise in the methods due to imaging and landmarking. Although the spread of landmarking repeats for some individuals was relatively larger than for others, it should be noted that landmarking of the model baseline and results would be more repeatable than landmarking of MR images as much of the intraindividual landmarking variation likely derived from the lower resolution of some scans which caused difficulty in accurately and repeatably identifying landmark points.

Other recent studies emerging in this field include Liang et al. (2024; 2023) who utilise a different tissue expansion method to analyse the stress and strain resulting from brain expansion on the sutures of the developing postnatal cranium. Our study instead focussed on the geometry of model deformation in response to tissue expansion, and included expansion of the masticatory muscles in addition to that of the brain. We also present our findings against a background of a real‐world normative ontogenetic series. For a more comprehensive review of sutures in response to tissue growth, see Liang et al. (2024). Other approaches to investigating the morphology of the cranium include Euclidean distance matrix analysis and anatomical network analysis (Bruner & Ripani, 2008; Schuurman & Bruner, 2024), high‐density network analysis (Goswami et al., 2019, 2023) and traditional cephalometrics and volumetrics (Jeffery et al., 2022; Jeffery & Manson, 2023).

Some future steps for this or similar finite element studies may involve the refinement of the model, particularly in the region of the face and cranial base, some of which was modelled *en bloc* which may affect the ability of the modelled basicranium to bend. Ontogenetic brain expansion is differential, with relatively more growth occurring in the frontal and occipital lobes of the cerebrum, and in the cerebellum, which more than doubles in size across postnatal ontogeny (Barbeito‐Andrés et al., 2020; Knickmeyer et al., 2008). Modelling this differential brain growth may allow future enhancement of this growth simulation allowing increased accuracy of biological replication; however, the additional model complexity may produce prohibitive computational load and simulation time. Increasing complexity of the model simulation will also result in increasingly complex simulation results which may prove difficult to analyse. The simpler the model, the clearer it will be to determine the cause of the observed effect; however, a model equally may fail to answer a particular question if it is too simplified, and therefore a balance of modelling complexity must be struck (Alexander, 2003). Material properties may be altered over simulation time in an attempt to better mimic the changing material properties throughout ontogeny, although more comprehensive material properties for human neonates and infants would need to be collected to allow this to be accurately modelled. Computational load and the time taken to run each simulation was a limiting factor in this analysis and constrained the growth simulations up to the equivalent of about 3–4 months postnatally. By utilising mesh reformatting, with associated additional computational load, future models may be able to simulate postnatal growth of soft tissues of the head beyond this timeframe, as in Jin et al. (2014) and Libby et al. (2017). Reformatting of the mesh would ensure that the model remained biologically accurate, and not just an extremely expanded neonatal cranium. The model may also be applicable in an evolutionary and/or interspecific context—by altering the rates and timings of growth of soft tissues, can another primate such as a macaque or an ancestral hominin be mimicked using the same starting base model? This may help to shed some light on the potential effects of prenatal versus postnatal growth—can a human neonate model be made to resemble a lesser primate or ancestral hominin, or are the differing rates of prenatal growth between species too great?

In summary, brain expansion exhibited the most influence on form of the cranial base and face in these simulations, which roughly aligned with normative data from individuals between 0 and 4 months of age. Shape changes associated with the brain growth simulation model included a relative ventral shift and reorientation of the anterior cranial base which agreed with the anticipated increase of cranial base flexion with increasing brain volume. Deepening of the posterior cranial fossa was observed, which would also aid in accommodating the growing brain. Expansion of masticatory muscles showed a lesser change in size of the basicranium and face. The simple finite element growth models utilised in this study mimicked biological growth reasonably well, which is suggestive of a largely structurally integrated relationship between regions of the developing head, particularly between the brain and basicranial‐facial complex within the developmental period studied here. However, there is ample scope for improvements and refinements to the finite element model which may result in more accurate results in the future, and potentially the ability to expand the model simulations into later postnatal ontogeny or into an evolutionary context.

## AUTHOR CONTRIBUTIONS

**Amy Manson:** acquisition of data, methodology, data analysis/interpretation, writing—original draft and writing—review and editing. **Nathan Jeffery:** conceptualization, methodology and writing—review and editing.

## Data Availability

All image data are available via the repositories listed within the Acknowledgements and Methods section.
